# Когнитивные и психоэмоциональные нарушения у женщин периода менопаузального перехода: возможности медикаментозной коррекции

**DOI:** 10.14341/probl13205

**Published:** 2023-02-25

**Authors:** С. А. Гаспарян, А. М. Чотчаева, С. М. Карпов

**Affiliations:** Ставропольский государственный медицинский университет; Ставропольский государственный медицинский университет; Ставропольский государственный медицинский университет

**Keywords:** гормональная терапия, менопауза, память, сон, депрессия, теория эуэстрогении, нейростероиды, когнитивная дисфункция

## Abstract

Увеличение численности населения старшей возрастной группы определяет возникновение новых вопросов, связанных с изучением возраст-ассоциированных заболеваний. В женском организме вступление в период менопаузального перехода является началом «старения» репродуктивной функции, что, в свою очередь, приводит к снижению уровней половых гормонов. Выявлена прямая связь между нарушениями соотношений эстрогенов, прогестерона, андрогенов и когнитивной активностью женщин. Данные анатомической локализации рецепторов половых гормонов, механизмы взаимодействия гормонов с этими рецепторами, пути реализации биологических эффектов объясняют действие стероидов на центральную нервную систему. Современные теории «здоровых клеток» и «эуэстрогении» объясняют влияния дополнительных критериев, таких как отсутствие неврологических заболеваний в анамнезе и длительность гипоэстрогении, на результат менопаузальной гормональной терапии (МГТ). К дополнительным факторам, способным повлиять на эффект МГТ, относятся состав гормональной терапии, дозы, методы введения, режимы (циклический, непрерывный), длительность лечения, наличие эндокринных заболеваний в анамнезе, в частности сахарного диабета, гинекологический анамнез (паритет, возраст менархе, применение комбинированных оральных контрацептивов), наследственность. В разделах изложены данные литературы о влиянии менопаузального перехода на развитие депрессии, изменения настроения, нарушения сна и ментальные способности. Также описываются данные современных исследований, направленных на объяснение отрицательных эффектов МГТ, описываемых в данных крупных рандомизированных исследований конца ХХ и начала ХХI вв. Противоречивость мнений современных исследователей, возможности нового прочтения результатов более ранних исследований подтверждают необходимость продолжения изучения данной темы.

## Abstract (en)

The increasing of older age group in the population determines studying of age related diseases and emergence of new investigations in this area. In Female body, entering the menopausal transition is the start of «aging» of reproductive function and linked with decreasing of sex hormons levels. A direct connection between changes of estrogen, progesterone, androgen ratios and cognitive function of women was revealed. The anatomical localization of sex hormone receptors, the mechanisms of interaction of hormones with these receptors determine the ways of implementing biological effects of steroids on the CNS. Modern theories of «healthy nerve cells» and «eu-estrogenemia» explains the role of additional criteria, such as the absence of neurological diseases history and the duration of hypoestrogenia, to the outcome of menopausal hormone therapy. Additional factors that can affect to MHT action include: the composition of hormone therapy, administration methods, regimens (cyclic, continuous), duration of treatment, history of endocrine diseases, diabetes mellitus, gynecological history (parity, menarche age, COC use), heredity. The sections present the effect of menopausal transition on the development of depression, mood changes, sleep disturbances and mental disabilities. The explanation of negative effects of menopausal hormone therapy to cognitive health is also described by modern point of view. The ambivalent opinions of researchers, the potential of new reading of the results of earlier studies, confirms the necessity of continuing study of this topic.

Возрастная структура мирового населения в ХХI в. претерпевает значительные изменения, связанные с увеличением численности населения старше 60 лет в 2,5 раза. Учитывая данную демографическую тенденцию, проблема снижения когнитивной функции и деменции среднего возраста становится все более актуальной [1]. ВОЗ определила целью тысячелетия концепцию политики «Активного долголетия», которая включает сохранение продуктивности и способности к аналитическому мышлению [2].

МКБ-10 включала в себя понятие «старческой астении», или «старческой хрупкости», основными клиническими проявлениями которой являлись снижение физической и когнитивной активности. Тогда как в МКБ-11 пересмотрено понятие старческой астении в пользу нового термина «aging-related disease» — заболевание, связанное со старением.

При этом известно, что старость — это закономерно наступающий заключительный этап онтогенеза, начало которого в женском организме отождествляется с вступлением в период менопаузы — «старения» репродуктивной системы, приводящей к снижению умственных способностей у женщин среднего возраста в том числе [3].

Дефицит половых гормонов, характерный для данного периода, является генетически детерминированной причиной структурных и функциональных изменений, свойственных стареющему организму [4]. Так, была доказана прямая связь между флюктуацией уровней половых гормонов и функциональной активностью ЦНС, приводящая к ухудшению когнитивной функции в перименопаузе, и это не случайное явление [5–7].

Ряд исследований, проведенных Европейским обществом по менопаузе и андропаузе, подтверждает, что менопаузальный период характеризуется снижением рабочей активности в 8,4 раза, а стресс на работе из-за менопаузальных симптомов (вазомоторных, когнитивных, урогенитальных) становится достаточно распространенной причиной для принятия решения о завершении карьеры.

Современные работодатели, идя в ногу с тенденциями общества и быстро интегрируя новые медиамодели в повседневную рабочую практику, требуют от сотрудников способностей к постоянному обучению новым технологиям, запоминанию и анализу большого количества информации. Угасание когнитивных способностей женщин в перименопаузе — периоде, когда она обладает всеми возможностями для активного продолжения своей карьеры, является одной из важнейших проблем, нуждающихся в социальной реализации [8].

Активная социальная роль является реальным фактором, способствующим сохранению когнитивной функции в пожилом возрасте [9].

Понятие менопаузального перехода включает период от 4–6 лет до последней менструации. В этот временной интервал у женщин среднего возраста наблюдаются прогрессирующее угасание активности яичников и физиологическая дисфункция гипоталамо-гипофизарной яичниковой системы, связанная с изменениями гормонального фона [10][11]. Впервые начинают проявляться менопаузальные симптомы: приливы, нарушение сна, перепады настроения, склонность к депрессиям, жалобы на ухудшение памяти, уменьшение скорости реакции, падение работоспособности, снижение либидо, урогенитальный дискомфорт [12]. Проявления неврологической симптоматики, такие как «туман в голове», бессонница и другие нарушения сна, депрессивность, ухудшение способности к познанию, являются одними из основных жалоб женщин в перименопаузе со стороны ЦНС и оказывают выраженное влияние на качество жизни пациенток, работоспособность и общее состояние здоровья [13].

Целью нашего обзора литературы явились попытка внести ясность в понимание механизма влияния менопаузального перехода на процесс ухудшения когнитивной функции, а также изучение способности менопаузальной гормональной терапии (МГТ) предотвратить или остановить эти изменения.

## МЕХАНИЗМ СТЕРОИД-ОПОСРЕДОВАННЫХ ИЗМЕНЕНИЙ В ЦНС

На протяжении различных жизненных этапов у женщин отмечаются выраженные изменения уровней половых гормонов: эстрогенов, прогестерона, андрогенов, от менархе до менопаузы. Эти изменения оказывают значительное воздействие на весь организм, в том числе на ЦНС, и могут быть причиной изменений настроения, работоспособности, поведения и познавательных возможностей [14].

В головном мозге обнаружено большое количество различных форм эстрогеновых рецепторов, которые особенно широко представлены в зонах, отвечающих за память и исполнительную когнитивную функцию, а также очень отличаются по проявлению эффектов [15].

Например, рецепторы эстрогена Еr-beta в основном представлены в коре головного мозга, а также в области гиппокампа, в то время как эстрогеновые рецепторы Еr-alpha больше всего представлены в холинергических нейронах базальных отделов переднего мозга. Интересным является мнение некоторых авторов о том, что ухудшение памяти и когнитивная дисфункция проявляются не только из-за уменьшения количества эстрогеновых рецепторов той или иной изоформы, но и за счет нарушений соотношения этих рецепторов [16].

Стероидные гормоны, влияющие на ЦНС, называются нейростероидами. Нейростероиды синтезируются как на периферии, так и в центральной/периферической нервной системе путем образования de novo из холестерина в нейронах и глиальных клетках. Также этот вид стероидов может быть продуктом местного метаболизма промежуточных стероидов, продуцируемых на периферии и способных к преодолению гематоэнцефалического барьера [14].

Эстрогены реализуют свой эффект посредством ряда нейротрансмиттеров, таких как норадреналин, глутамат, ацетилхолин, серотонин. Холинергическая нейротрансмиттерная система осуществляет влияние эстрогена на состояние памяти. Эстроген обладает нейропротекторным и нейротрофическим эффектами за счет усиления синаптической пластичности, роста нейронов, активации нейрогенеза в гиппокампе, а также защищая нейроны от повреждения и апоптоза. Кроме того, выявлена способность эстрогена к усилению синтеза аденозинтрифосфата и митохондриальному дыханию, что крайне важно для столь энергозатратного органа, как мозг. Доказано антиоксидантное действие эстрогена в головном мозге за счет ингибирования синтеза свободных радикалов и усиления репарации ДНК [14].

Клинические исследования влияния эстрадиола продемонстрировали усиление роста и дифференцировки нервных клеток за счет геномного процессинга и влияния на факторы роста. Также в исследовании эффектов гормональной терапии была обнаружена способность к повышению плотности дендритных шипов и формирования новых синапсов в поле Cornu Ammonis (СА1) гиппокампа и в префронтальном отделе коры головного мозга [14].

Половые гормоны реализуют свои эффекты на мозг в корковых и в подкорковых структурах через геномные и негеномные пути [17].

Большинство исследований с доказанной эффективностью в данной области проведено на животных, однако считается, что ряд молекулярных и клеточных процессов, осуществляемых посредством модуляции экспрессии генов и активации сигнальных путей в нервной системе, применим и к людям. Половые гормоны осуществляют свое действие через ядерные, мембранные, синаптические рецепторы, а также рецепторы, локализованные в дендритных шипиках, митохондриях. Подобные рецепторы были идентифицированы в клетках глии, обеспечивающих регуляцию синтеза миелина и, соответственно, потенциально играющих значительную роль в патогенезе демиелинизирующих заболеваний [18]. Была обнаружена протективная способность эстрадиола в отношении нейродегенерации при болезни Альцгеймера, являющейся на данный момент наиболее распространенной причиной деменции [18–20]. Подобный эффект связан с противовоспалительными свойствами эстрадиола, участвующего в клиренсе амилоида Beta. Предотвращая отложение этого агрессивного белка, являющегося одной из основных причин нейродегенерации, наравне с хроническим воспалением и митохондриальной дисфункцией, эстрадиол ингибирует преждевременные признаки ментального старения [21].

Противовоспалительный эффект эстрогенов проявляется в снижении нейроинфламмации и индуцировании нейроиммуномодуляции, а также нейропротекции в глиальной ткани [22, 23].

Эстрогены и андрогены имеют общие метаболические пути и функциональные свойства. Имеются исследования, подтверждающие благотворный нейропротективный эффект андрогенов на головной мозг, реализуемый при взаимодействии с инсулиноподобным фактором роста 1-го типа.

Другой компонент МГТ — прогестаген также является нейростероидом. Прогестерон — это мощный регулятор нейрогенеза, влияющий на большинство биоэнергетических систем организма. Этот нейростероид действует как по классическому — рецепторному пути, регулируя транскрипцию генов, так и по неклассическому — сигнальному, активируя сигнальные каскады и транскрипцию различных генов [14].

Главными эффектами обоих путей являются нейропролиферативный, антиапоптотический, а также биоэнергетическая регуляция. Прогестерон производится как гонадами, так и олигодендроцитами, которые трансформируют его в дегидропрогестерон и аллопрегнанолон, регулирующие миелинизацию и моделирующие ГАМК-эргические рецепторы [24].

Несмотря на то что прогестерон, как и эстрадиол, способен к синаптогенезу и увеличению плотности дендритных шипиков, он не обладает долговременным потенцирующим эффектом в поле СА1, что объясняет отсутствие эффекта на формирование эпизодической памяти (в отличие от эстрогена) [14].

Анализ данных современной литературы продемонстрировал возможности непосредственного позитивного влияния половых стероидных гормонов на нейротрофику, нейропролиферацию и нейропротекцию, что позволяет нам рассматривать МГТ как эффективный превентивный и терапевтический метод, направленный на коррекцию когнитивных изменений периода менопаузального перехода.

## ПЕРИМЕНОПАУЗА И КОГНИТИВ

В зарубежной литературе часто встречается описание когнитивной дисфункции в период менопаузального перехода образным выражением «тумана в голове» (brain fog) [25]. Выявлена прямая зависимость между частотой жалоб на ухудшение когнитивной функции (ухудшение результатов тестов на внимание, словесную-вербальную, оперативную-краткосрочную память) и длительностью менопаузы [26]. Отмечается влияние менопаузального статуса на развитие таких симптомов, как беспричинная тревога, депрессивное настроение, беспокойство, нарушения памяти и дисфункция сна [27][28].

Ряд исследователей описывают значимое снижение памяти и скорости когнитивных процессов, начиная с переходного периода, вследствие не только органических изменений в ЦНС, но и негативного влияния вазомоторных симптомов, дисфункции сна, психоэмоциональных нарушений [9].

Все большую актуальность приобретает теория перименопаузального «критического окна». В основе данной теории лежит современная концепция «эуэстрогении», согласно которой раннее инициирование гормональной терапии в перименопаузальный период способно нивелировать отрицательное влияние репродуктивного старения на когнитив [21].

Дело в том, что эстрогеновые рецепторы, широко представленные в большинстве органов и тканей (подтверждено около 3600 эстрогеновых сигнальных путей), хорошо отвечают на прием эстрогенов, назначенных после краткого периода абстиненции, однако после продолжительного «БЕЗДЕЙСТВИЯ» в условиях гипоэстрогенемии так называемая «ре-эстрогенизация» с помощью экзогенных эстрогенов может оказаться невозможной [15][29].

Бόльшая подверженность риску развития деменции женщин по сравнению с мужчинами старшей возрастной группы доказана рядом клинических исследований. Проведенное относительно недавно перекрестное исследование, включавшее женщин ранней возрастной группы, выявило лучшую результативность женщин в задачах на детальную память по сравнению с мужчинами того же возраста [16]. Однако эти половые различия сглаживались в постменопаузальном периоде [30][31].

По данным ряда современных авторов [14], у пациенток с более высоким уровнем эстрадиола в плазме отмечалась лучшая работоспособность. Другие же исследования не продемонстрировали никакой связи между познанием, когнитивной функцией и уровнями эстрогенов [32]. Но в данном аспекте важно отметить факторы, изменяющие метаболический эффект эстрогена, оказываемый на ЦНС [14]. К таким факторам относятся теории «здоровых клеток» и «критического окна».

## ТЕОРИЯ «ЗДОРОВЫХ КЛЕТОК»

Суть данной теории заключается в том, что поддержание гликолитического метаболизма экзогенным эстрогеном возможно только в здоровых нервных клетках, а в условиях нейродегенерации патологическая нагрузка на измененные клетки усугубляется, что приводит к негативным результатам применения эстрогеновой/эстроген-гестагеновой терапии.

В клинических моделях окислительного стресса, нейротоксичности, ишемии и апоптоза с использованием здоровых нервных тканей (без предварительного моделирования нейродегенеративных патологических изменений) ряд исследований отмечает нейропротективный эффект эстрадиола [2].

## ТЕОРИЯ «КРИТИЧЕСКОГО ОКНА»

Концепция данной теории основана на понятии о «эуэстрогении», которая объясняет лучший ответ на гормональную терапию, инициированную до момента наступления выраженного эстрогенного дефицита, например, в периоде менопаузального перехода. Установлено, что более длительное воздействие эндогенных эстрогенов может быть связано с лучшими когнитивными способностями женщин старшего возраста.

Недавнее клиническое исследование 1315 женщин продемонстрировало прямую корреляцию показателей вербальной памяти с продолжительностью фертильного периода (чем больше возраст естественной или хирургической менопаузы, тем лучше показатели вербальной памяти) [17].

В другом исследовании, включившем 3602 женщины, более длительный период фертильности не был связан со снижением риска деменции.

Несмотря на глубокую связь между половыми гормонами и когнитивной функцией, эффект заместительной гормональной терапии на способность к познанию, а также нейропротекторные, нейротрофические и нейропролиферативные действия гормонов все еще остаются противоречивыми. Существует ряд исследований, исключающих когнитивные преимущества эстрогеновой или комбинированной эстроген-гестагенной терапии у пациенток старше 65 лет в поздней постменопаузе без сопутствующей деменции.

Несмотря на распространенность жалоб на ухудшение памяти в перименопаузальном периоде, результатов продолжительных исследований, демонстрирующих влияние гормональных изменений на когнитивную функцию, недостаточно [14].

Большая часть лонгитюдных исследований когнитивной дисфункции была сосредоточена на пациентах старше 65 лет. Только два продолжительных когортных исследования продемонстрировали результаты для женщин, переживающих менопаузу, это исследования QIWI и SWAN.

Согласно обзору G. Greendale и соавт. (2011), в обоих исследованиях отмечалось значительное снижение когнитивной функции, особенно в перименопаузе. Об этом свидетельствует снижение «эффекта обучения» при когнитивной оценке в динамике, а не снижение когнитивных способностей [18].

В первом исследовании, QIWI, наблюдение проводилось за женщинами в пременопаузальном периоде в течение 18 мес.

Во втором исследовании, SWAN, наблюдение проводилось за женщинами в пери- и постменопаузе в течение 4 лет. Анализ данных результатов исследований направлен на устранение пробелов в нашем понимании когнитивных эффектов гормональной терапии в перименопаузальном периоде. Исследование включило 2362 пациентки в пременопаузе, ранней перименопаузе и постменопаузе. В течение 4 лет проводилась оценка когнитивной активности на фоне МГТ по следующим показателям: скорость обработки, SDMT (тест модальности символьных цифр), вербальная память (EVMT-тест памяти восточного Бостона) и рабочая память (размах цифр в обратном направлении). По результатам данного исследования отмечались более высокие показатели при повторном определении SDMT у пациенток, начавших терапию в пременопаузе, ранней пери- и постменопаузе, в то время как аналогичные показатели пациенток в поздней постменопаузе никак не улучшались. Показатели EVMT улучшались у пациенток в период пре- и постменопаузы, но не увеличивались во время ранней и поздней перименопаузы. По шкалам SDMT и EVMT пациентки в постменопаузе, принимающие гормональную терапию, продемонстрировали более низкие когнитивные способности по сравнению с когортой пациенток в пременопаузе, но показатели пациенток в постменопаузе, не получающих гормональную терапию, не отличались от показателей пациенток в пременопаузе. По результатам данного исследования, начало приема гормонов до прекращения менструации имело положительный эффект, в то время как инициирование гормональной терапии в постменопаузе оказывало отрицательное влияние на когнитивные функции [18].

Исследование SWAN проводится с 1996 г. В нем принимали участие женщины на протяжении всего менопаузального перехода. В ближайшее время ожидаются новые результаты данного лонгитюдного исследования, которые позволят нам сделать выводы о долгосрочных последствиях гормональных изменений у женщин среднего возраста.

В то время как множественные фундаментальные научные труды выявляли многообещающие результаты эффективного влияния гормональной терапии на предотвращение когнитивной дисфункции, ряд клинических испытаний, таких как WHI (инициатива женского здоровья) и WHIMS (исследование памяти инициативы женского здоровья), выявили значительные риски, в том числе отрицательный эффект на когнитивную функцию, связанный с МГТ [21].

Исследование WHI. Исследование проводилось с 1993 по 1998 гг., в нем приняли участие 161 809 женщин в возрасте от 50 до 79 лет. В испытании гормональной терапии более 27 000 женщин были разделены на три группы, получавшие перорально: 1) комбинацию эстрогена (CEE, конъюгированный лошадиный эстроген) и прогестерона (MПА, медроксипрогестерона ацетат); 2) только СЕЕ (при гистерэктомии); 3) плацебо. Исследование проводилось в течение 5,2 года, после чего было прекращено из-за неблагоприятных исходов, а именно повышения частоты инвазивного рака молочной железы в 1-й группе и повышения риска инсультов и тромбозов в 1-й и 2-й группах.

Исследование WHIMS. Данное исследование включало 4532 женщины в постменопаузе из исследования гормональной терапии WHI, которым на момент начала исследования было 65 лет и более и у которых не было клинических признаков деменции. Женщины в данном исследовании каждые полгода проходили когнитивные опросники и ежегодно — когнитивные тесты. Результаты этого исследования продемонстрировали, что пациентки из 1-й группы WHI обеспечивались минимальной защитой когнитивных функций, а скорее, МГТ повысила вероятность развития деменции. Данные результаты отмечались не только клинически, но и инструментально. Более детальное исследование в рамках WHIMS, включающее в себя сравнительный анализ результатов МРТ у пациентов из исследуемых клинических групп, ранее получавших гормональную терапию в рамках WHI, выявило снижение объема гиппокампа, лобных долей и всего мозга, хотя не было представлено доказательств, позволяющих предположить различия между группами в ишемической нагрузке. Также необходимо отметить, что на исходном этапе данного исследования было обнаружено, что повышенная нагрузка на белое вещество и более низкие когнитивные функции являются предикторами неблагоприятного эффекта гормональной терапии, выражающегося в усугублении когнитивной дисфункции и снижении неврологического статуса. Важно отметить, что все пациентки получали лечение спустя несколько лет после менопаузального перехода [14].

При вторичном анализе данных WHIMS (WHIMS-Y, исследование памяти в рамках инициатив женского здоровья — молодые) были проанализированы 1326 пациенток от 50 до 55 лет из исследования WHI, которые начали принимать гормональную терапию вскоре после менопаузы. М. Espeland и соавт. (2013) показали, что у этой группы пациентов не наблюдалось ухудшения или статистически значимого улучшения когнитивной функции в течение следующих нескольких лет после прекращения МГТ. У пациенток, принимавших МГТ в возрасте 65–79 лет, отмечалось значимое снижение когнитивного статуса, памяти и работоспособности [22].

Последующие исследования продемонстрировали, что основной причиной столь противоположных результатов является различие в возрасте начала гормональной терапии, что подтверждает теорию «критического окна», в которой время инициирования гормональной терапии является ключевым фактором, обеспечивающим положительный эффект лечения.

Так, пациентки, принимающие МГТ в перименопаузальном периоде, демонстрировали лучшие показатели вербальной памяти по сравнению с женщинами, не получавшими МГТ, у которых отмечалось прогрессирующее снижение работоспособности (результаты оценивались через несколько лет спустя после лечения). Схожие результаты демонстрировались в исследовании женщин, использовавших МГТ непосредственно после овариэктомии.

В исследовании, включавшем 428 пациенток, начавших гормональную терапию в возрасте до 56 лет, были продемонстрированы результаты, превосходящие таковые у пациенток, начавших применение МГТ после 56 лет, при оценке общих когнитивных показателей, скорости психомоторной реакции, беглости речи. Вторая группа пациенток данного исследования, получающих гормональную терапию позже 56 лет, продемонстрировала ухудшение способности к познанию по сравнению с контрольной группой пациенток, не получавших МГТ.

Другие данные, сравнивающие пациенток в перименопаузальном периоде со средним возрастом 48,8 года, начавших лечение и получавших его в течение 5,2 года, с пациентками, использующими гормональную терапию в течение 14,3 года, а также с контрольной группой пациенток, никогда не применявших МГТ, продемонстрировали явный положительный эффект у 1-й группы по сравнению с контролем по показателям умственной гибкости, когнитивному функционированию [14].

Ряд клинических исследований продемонстрировал преимущество раннего начала гормональной терапии, инициированного непосредственно перед переходом в менопаузу, в сравнении с постменопаузой.

Опубликованы исследования, в которых [14] было отчетливо видно снижение частоты когнитивной дисфункции при инициировании гормональной терапии в рамках первых 2–3 лет после перехода к менопаузе, а при последующем обследовании этих пациенток спустя 5 и 15 лет нарушение когнитивных функций отмечалось на 64% реже.

При оценке беглости речи и состояния вербальной памяти у 727 женщин, начавших гормональную терапию вскоре после наступления менопаузы, результаты были значительно лучше, чем у пациенток, не использовавших гормональную терапию.

Все описанные выше исследования подтверждают гипотезу «критического окна», демонстрирующую прямую зависимость результатов лечения от времени старта МГТ. Следовательно, старт гормональной терапии должен соответствовать периоду менопаузального перехода, не дожидаясь симптомов «истощения эстрогенов».

Ряд исследователей оценивают время старта МГТ как вторичный фактор по отношению к теории «здоровых клеток» (неизмененной митохондриальной биоэнергетики), ориентируясь на клинические и инструментальные результаты WHIMS у пациенток старше 65 лет [33][34].

Отрицательные результаты в виде снижения показателей вербальной памяти даже при начале гормональной терапии вскоре после менопаузы (в среднем 52 года) у пациенток из исследования WHIMS, получавших комбинацию эстрогена СЕЕ и синтетического прогестина МПА, в работе Нильсона и Бринтона объясняются антагонизмом МПА, противодействующим нейропротекторному эффекту СЕЕ. Ими было проведено исследование in vitro, по результатам которого было выявлено неблагоприятное воздействие комбинации СЕЕ+МПА на нейроны гиппокампа, способствующее ухудшению вербальной памяти. Так, было продемонстрировано влияние состава гормональной терапии на результат лечения наравне с путем введения и схемой приема (циклическая/непрерывная МГТ).

Изучая данные полученных результатов, большинство исследователей пришли к выводу о необходимости более тщательного подбора МГТ, компоненты которой не будут обладать антагонизмом, а тем более отрицательным влиянием на способность к познанию и показатели памяти. С данной целью возможно применение биохимически нейтральных прогестагенных компонентов, например дидрогестерона, который за счет своего селективного влияния на рецепторы способен изменять ментальные функции и свободно реализовывать свой нейропротективный эффект путем регуляции митогенактивированной протеинкиназы, активируя сигнальные пути нейротрофического фактора головного мозга (BDNF), который модулирует метаболизм нейронов [14].

Было показано, что все описанные выше факторы влияют на результаты лечения независимо от сроков. В крупных исследованиях ELITE и WISH были выявлены дополнительные данные репродуктивного анамнеза, способные к искажению результатов эффекта гормональной терапии. К таким факторам относятся: возраст менархе, количество беременностей, возраст, при котором наступила последняя беременность, продолжительность репродуктивного периода, применение комбинированных оральных контрацептивов в анамнезе. Все вышеописанное положительно коррелирует с когнитивным статусом в пожилом возрасте [35].

Формирование теорий «критического окна», «здоровых клеток», а также открытие множества факторов, влияющих на результат МГТ, стимулировало исследователей к дальнейшему поиску возможных показателей, искажающих эффекты гормональной терапии.

В новой работе, посвященной влиянию соматических заболеваний на результат гормональной терапии, М. Espeland и соавт. [22] выявили вариабельность данных WHIMS и прямую зависимость от наличия у пациентки сахарного диабета 2-го типа. Так, пациентки с сахарным диабетом 2-го типа, применяющие только эстрогены, имели более высокий риск когнитивной дисфункции и возможной деменции в сравнении с той же возрастной группой с/без диабета в анамнезе, не получавших гормональную терапию. Не было отмечено связи наличия сердечно-сосудистых заболеваний, ожирения, артериальной гипертензии в анамнезе с первичным когнитивным статусом. Результаты исследований сохранялись в течение 10 лет после прекращения терапии.

М. Espeland и соавт. [22] утверждают, что пациентки, имеющие метаболическую зависимость от кетогенеза (тип метаболизма, развивающийся при сахарном диабете), испытывают усугубление метаболической дисрегуляции при МГТ. Экзогенные эстрогены, вероятно, подавляют сформированные кетогенные процессы, при том, что гликолитический метаболизм еще не смог восстановиться, что и приводит к усугублению биоэнергетической дисрегуляции. Пациентки из 1-й группы (СЕЕ+МПА) с наличием сахарного диабета, скорее всего, не демонстрировали выраженной когнитивной дисфункции за счет антагонистического эффекта МПА, подавляющего влияние эстрогена на кетогенный метаболизм.

Для объяснения столь противоречивых результатов и разрешения сохраняющихся разногласий в научной литературе по поводу преимуществ и рисков гормональной терапии на когнитивный статус было разработано исследование KEEPS-Cog.

KEEPS-Cog — это вспомогательное исследование большого научного труда KEEPS (изучение эффектов гормональной терапии на сердечно-сосудистую систему), разработанное для подробного анализа изменений в познании и настроении на фоне старта гормональной терапии в период менопаузального перехода. Результаты KEEPS-Cog были аналогичны WHIMS-Y [33].

В исследовании KEEPS-Cog приняли участие 662 женщины, применяющие комбинацию: 1) трансдермальный эстрадиол и микронизированный прогестерон; 2) низкие дозы СЕЕ и микронизированный прогестерон; 3) плацебо. Старт гормональной терапии у всех этих женщин был в раннем менопаузальном периоде, а результаты не продемонстрировали статистически значимого увеличения или снижения показателей памяти, концентрации внимания по сравнению с плацебо, но в то же время пациентки из 2-й группы, принимавшие пероральный СЕЕ, продемонстрировали снижение симптомов депрессии и тревоги в течение следующих 4 лет. Все пациентки данного исследования не подвергались ни гистерэктомии, ни оофорэктомии. Исследователи KEEPS-Cog применяли трансдермальную форму эстрогена, а также более низкую СЕЕ, чем исследователи WHIMS. Режим введения препаратов был циклическим для максимального приближения к естественным циклическим изменениям женского организма, в то время как в WHIMS использовали непрерывное введение MПА [36].

Описанные выше ключевые различия в дизайне исследования, выборке пациенток, вероятно, способствовали расхождению результатов между KEEPS-Cog и WHIMS. Планируется повторная оценка участников исследования KEEPS в новом исследовании KEEPS-Continuation Study, которое будет проведено спустя 10 лет от KEEPS. В нем планируется оценить лонгитюдные когнитивные и психоэмоциональные эффекты МГТ, а также корреляты нейровизуализации мозга и наличие биомаркеров риска болезни Альцгеймера.

Еще одно крупное рандомизированное исследование ELITE сравнивало результаты раннего и более позднего старта МГТ. Оно включало пациенток следующих возрастных групп: 1) в пределах первых 6 лет от менопаузы; 2) от 10 лет и более в постменопаузе. Все пациентки получали пероральный эстрадиол в течение 5 лет. Результаты не показали улучшения или ухудшения когнитивных функций у молодых женщин. Но, в отличие от данных WHIMS, у пациенток в поздней возрастной группе (более 10 лет после менопаузы) не было выявлено отрицательного воздействия на когнитивную функцию [14].

Результаты крупного рандомизированного «исследования здоровья медсестер», включившего 13 807 женщин от 70 до 80 лет, подтвердили теорию «критического окна». При длительном применении эстрогенов (не менее 5 лет) или комбинации эстрогена и прогестерона результаты большинства тестов на оценку когнитивных функций значительно ухудшались по сравнению с пациентами, не применявшими гормональную терапию [14].

Несмотря на противоречивость и неоднозначность результатов современных исследований, большинство ученых сходятся во мнении о благоприятном воздействии раннего старта МГТ у пациенток с первичными признаками гипоэстрогении, такими как ночная потливость, вазомоторные симптомы, нарушения сна, ухудшающими качество жизни.

## ПЕРИМЕНОПАУЗА И ПСИХОЭМОЦИОНАЛЬНЫЕ ИЗМЕНЕНИЯ

На протяжении жизни женщины более подвержены депрессиям и эмоциональной уязвимости, особенно в такие периоды, как лютеиновая фаза менструального цикла, беременность, послеродовый период и менопаузальный переход. Депрессия, перепады настроения, бессонница, ночные пробуждения, ранние пробуждения, скорее всего, тесно связанные между собой, требуют подробного изучения и специального лечения [37–39].

Современные исследования показывают, что на уровень настроения в перименопаузе может влиять множество других факторов, таких как психосоциальные, общесоматические, демографические, уровень образования, трудоустроенность, семейное положение, курение, бездетность, тревожность, предменструальный синдром, эпизоды послеродовой депрессии в анамнезе, стрессовые жизненные события (смерть партнера или близких родственников), индекс массы тела, самооценка (неудовлетворенность своей внешностью и качеством жизни очень распространены у женщин в периоде менопаузального перехода) [37][38].

Доказано влияние возраста, индекса массы тела, уровня физической активности и других факторов образа жизни на интенсивность симптомов менопаузы, а именно на психоэмоциональное состояние женщин. Уровень самооценки обратно коррелирует с уровнем депрессии и тревогой, а также рядом вазомоторных симптомов [14].

Несмотря на отсутствие полной ясности механизма влияния эстрогенов на психоэмоциональное состояние в перименопаузальном периоде, следует учитывать их роль на развитие перименопаузальной депрессии [40].

Как указано ранее, эстрадиол (Е2) обладает способностью к регуляции синтеза, метаболизма и проявления эффектов классических нейротрансмиттеров, таких как серотонин, дофамин и норадреналин. Также выявлено стимулирующее действие эстрогена на нейротрофический фактор головного мозга (BDNF), являющийся важным нейропротекторным веществом, дефицит которого доказан в патогенезе депрессии [41].

Прогестерон и его метаболит аллопрегненалон способны ингибировать синаптическую передачу, стимулируя ГАМК-эргическую систему и реализуя тем самым анксиолитический эффект, схожий с действием бензодиазепинов. У людей понижение уровня аллопрегненалона связано с депрессией. Негативное влияние на когнитивные способности женщин, выявленное в ряде исследований, объясняется ГАМК-эргическим эффектом прогестерона на гиппокамп [14].

Австралийское продолжительное исследование женского здоровья, включившее 5336 женщин, продемонстрировало зависимость риска возникновения депрессии от наличия и объема перенесенного оперативного вмешательства на репродуктивных органах. Так, у пациенток с гистерэктомией и оофорэктомией риск развития депрессии был на 44% процента выше, чем у пациенток с интактной маткой и яичниками (контрольная группа). У пациенток с гистерэктомией и интактными яичниками риск возникновения депрессивного синдрома на 20% выше, чем у пациенток из контрольной группы [42].

Аналогичные результаты повышения риска депрессии в период менопаузального перехода были получены при анализе данных других крупных продолжительных исследований. Например, Гарвардское исследование настроения и циклов среднего возраста, Австралийское исследование женского здоровья, Сиэтлское исследование здоровья женщин среднего возраста [43]. Проанализировав данные этих исследований, J.L. Gordon и соавт. [44] подчеркивают, что в патогенезе большого депрессивного расстройства большее значение имеет флюктуация половых гормонов, а не их уровень.

Было проведено исследование, в котором приняли участие 203 женщины в пременопаузе, наблюдавшиеся в течение 14 лет и прошедшие естественную менопаузу. Результаты его продемонстрировали, что риск депрессивных симптомов был значительно выше в переходный период менопаузы по сравнению с постменопаузой [14]. Это и другие исследования позволили сформировать гипотезу Penn Ovarian Aging Study (POAS), заключающуюся в том, что выраженность депрессивных симптомов увеличивается в перименопаузе и снижается в течение нескольких лет после менопаузы.

В одном из рандомизированных исследований оценивалось влияние МГТ на риск возникновения депрессивного расстройства в перименопаузальном и раннем постменопаузальном периодах. Работа включала в себя 172 женщины, разделенные на две группы: 1) получали трансдермальный Е2 в сочетании с пероральным микронизированным прогестероном в циклическом режиме, 2) группа плацебо-контроля. В результате пациентки из 1-й группы проявляли значительно меньший риск развития депрессивных симптомов по сравнению с участницами из контрольной группы, получавшими плацебо, в соотношении 32,3%/17,3% соответственно [44][45]. Этот эффект больше проявлялся при старте терапии в раннюю перименопаузальную стадию [38].

Как следует из недавно опубликованных рекомендаций Р. Maki и соавт. [46], несмотря на способность эстрогенов усиливать реакцию на антидепрессанты у пациенток среднего и пожилого возраста, назначать МГТ все же рекомендуется при наличии других симптомов менопаузы, например вазомоторных.

Различия в методологии, используемой для оценки репродуктивной стадии психического состояния, противоречивы в различных исследованиях и влияют на объективность анализа результатов. Большая часть литературы о психическом здоровье женщин рассматривает период менопаузального перехода как фактор риска развития депрессивного расстройства [37].

Опубликованы данные исследования, согласно которым не удалось выявить каких-либо статистически значимых эффектов применения гормональной терапии у женщин в постменопаузе, не страдающих депрессией.

В трудах ряда современных авторов [14] также не было продемонстрировано значимых изменений после 20 нед применения перорального Е2 по сравнению с плацебо у пациенток, не страдающих депрессией перименопаузального возраста.

Анализ результатов исследований, схожих с указанными выше, показал, что не рекомендуется превентивный прием МГТ без симптомов депрессии и нарушения настроения, несмотря на очевидное благотворное влияние на психоэмоциональное состояние женщин в перименопаузе. При наличии у пациентки депрессивных симптомов, а также других сопутствующих симптомов менопаузы, например вазомоторных, МГТ окажет положительный эффект, в том числе за счет способности потенцирования эффекта антидепрессантов [47].

При решении вопроса терапии депрессивного расстройства пациенток в менопаузе необходимо точно установить этиологию депрессивных симптомов для дифференцировки пациенток с эпизодами депрессий в анамнезе от пациенток с депрессией менопаузального перехода. В первом случае пациенткам рекомендуется терапия антидепрессантами как препаратами первой линии [47]. Для 2-й группы пациенток возможно рассмотрение гормональной терапии наравне с немедикаментозными методами лечения (например, физические упражнения, правильное питание и здоровый сон) [48].

## НАРУШЕНИЕ СНА В ПЕРИОД МЕНОПАУЗАЛЬНОГО ПЕРЕХОДА

Женщины среднего возраста в перименопаузе часто жалуются на нарушение качества сна, уменьшение длительности сна, прерывистый сон и ночное апноэ. Начавшись в перименопаузе, эти жалобы усугубляются в постменопаузальном периоде [49].

Нарушение сна остается серьезным фактором риска развития патологии сердечно-сосудистой системы, сахарного диабета, метаболического синдрома и ожирения, а также психоэмоциональной дисфункции, что приводит к снижению качества жизни и работоспособности [2].

Проблемы со сном можно разделить на три группы: трудности с засыпанием, многократные ночные пробуждения/прерывистый сон и ранние пробуждения. Продолжительное исследование более чем 3000 женщин перименопаузального периода на протяжении 8 лет продемонстрировало, что прерывистый сон и ночные пробуждения были наиболее ранней и распространенной жалобой [50][51].

Интересные данные получены в отношении влияния вида (хирургическая/естественная) менопаузы на нарушения сна. Так, хирургическая менопауза связана с более серьезными нарушениями сна, чем естественная, а риск развития тяжелых вазомоторных симптомов значительно выше у пациенток после двусторонней оофорэктомии в сравнении с женщинами с естественной менопаузой.

Большинство исследований указывает на тесную связь нарушений сна с вазомоторными симптомами. Данные исследования SWAN продемонстрировали, что пациентки с умеренными и тяжелыми приливами (частота 6–14 в сутки) почти в 3 раза чаще страдают от ночных пробуждений в отличие от не имеющих их [14].

На качество сна в перименопаузальном периоде также оказывают влияние другие проблемы: ожирение, эндокринопатии, заболевания сердечно-сосудистой системы, желудочно-кишечного тракта, нарушения мочеиспускания, хронические болевые синдромы, курение, злоупотребление кофеином, использование нейроактивных препаратов, селективных ингибиторов обратного захвата серотонина, бронходилататоров, противоэпилептических средств, заместительная гормональная терапия при нарушениях функции щитовидной железы [49].

Доказано влияние комбинированной МГТ на улучшение качества сна как у пациенток с вазомоторными симптомами (за счет уменьшения ночного потоотделения и др.), так и без вазомоторных симптомов (за счет ГАМК-эргического седативного эффекта прогестагенов).

## ЗАКЛЮЧЕНИЕ

Таким образом, анализ данных литературы продемонстрировал полярность мнений современных исследователей относительно влияния МГТ на когнитивные функции женского организма в периоде менопаузального перехода, исходя из концепции «эуэстрогенемии». Это требует дальнейших продолжительных наблюдений за эффектами гормональной терапии с учетом возраста начала старта, состава, доз и схем применения препарата, тщательного анализа анамнестических данных и объективной оценки исходного соматического и когнитивного состояния женщины.

## ДОПОЛНИТЕЛЬНАЯ ИНФОРМАЦИЯ

Источники финансирования: Работа выполнена по инициативе авторов без привлечения финансирования.

Конфликт интересов. Авторы декларируют отсутствие явных и потенциальных конфликтов интересов, связанных с содержанием настоящей статьи.

Участие авторов. Гаспарян С.А. — существенный вклад в концепцию и дизайн исследования, внесение в рукопись существенной правки с целью повышения научной ценности статьи; Чотчаева А.М. — существенный вклад в получение и анализ данных, интерпретацию результатов, написание статьи; Карпов С.М. — существенный вклад в концепцию и дизайн исследования, внесение в рукопись важной правки с целью повышения научной ценности статьи. Все авторы одобрили финальную версию статьи перед публикацией, выразили согласие нести ответственность за все аспекты работы, подразумевающую надлежащее изучение и решение вопросов, связанных с точностью или добросовестностью любой части работы.
